# Chemical Composition of Volatile Extracts from Black Raspberries, Blueberries, and Blackberries and Their Antiproliferative Effect on A549 Non-Small-Cell Lung Cancer Cells

**DOI:** 10.3390/life12122056

**Published:** 2022-12-08

**Authors:** Inah Gu, Cindi Brownmiller, Luke Howard, Sun-Ok Lee

**Affiliations:** Department of Food Science, University of Arkansas, Fayetteville, AR 72704, USA

**Keywords:** berry volatiles, composition, antiproliferative effect, apoptosis, lung cancer, blackberry, black raspberry, blueberry

## Abstract

Berry volatiles are responsible for the berry aroma but there is limited information available on the health-promoting activities of berry volatiles. The objectives of this study were to evaluate the chemical composition of volatile extracts from black raspberries, blueberries, and blackberries and investigate their antiproliferative effect and apoptotic mechanisms on A549 lung cancer cells. The chemical composition of three berry volatile extracts (BVEs) was identified by using gas chromatography-mass spectrometry. Cells were treated with different dilutions of three BVEs for 48 h and determined for cell proliferation and apoptosis. Total volatiles in BVEs were 1.6–3.2 mg/L. Two-fold diluted BVEs significantly inhibited cell proliferation after 48 h, inducing apoptosis (*p* < 0.05). Blackberry volatile extract significantly reduced the inactive form of apoptotic proteins, including poly adenosine diphosphate-ribose polymerase (PARP), procaspase-9, and procaspase-3 compared to the control (*p* < 0.05). Blueberry volatile extract showed higher apoptotic cell death (*p* < 0.05) with a slightly higher cell population in G0/G1 phase than other berries. These results showed that volatile extracts from three berries have the antiproliferative effect on human lung adenocarcinoma cells partially via apoptosis, suggesting that volatiles from three berries may have potential anti-cancer activity through apoptosis in lung cancer.

## 1. Introduction

Lung cancer is one of the most common cancers and the leading cause of death that are related to cancer [1]. The American Cancer Society estimated that there were about 0.2 million new cases and 0.1 million deaths from lung cancer in the USA in 2022 [2]. There are two major types of lung cancer: non-small cell lung cancer (NSCLC) and small cell lung cancer (SCLC). Non-small cell lung cancer accounts for 85% of all lung cancer and includes squamous cell carcinoma, large cell carcinoma, and adenocarcinoma [3,4]. Tobacco smoking is a major risk of lung cancer, but also passive smoking, alcohol, air pollution, occupational exposure, and genetic factor affect lung cancer [3,5]. Since it is hard to detect or diagnose lung cancer at an early stage, lung cancer has a high mortality rate [6]. Currently, chemotherapy, radiotherapy, surgery, and immunotherapy have been used for lung cancer treatment, but they lack effectiveness and have increased drug resistance [7]. Thus, more research is needed to support the current treatment methods for lung cancer.

Apoptosis is regulated cell death by intracellular caspases causing deoxyribonucleic acid (DNA) fragmentation and nuclear shrinkage [8]. Apoptosis is necessary for developing and remodeling tissues in particular spaces, including the interdigital space of a hand and foot during early development [9]. In adults, apoptosis is mostly used in the destruction of damaged cells that are unrepairable. While necrosis disrupts the cell membrane and releases intracellular products to extracellular space, apoptosis condenses nuclear and intracellular products and does not release them outside of cells [10]. Thus, apoptosis has an important role in preventing cancer, and apoptosis of cancer cells has become one of the promising targets for treatment agents. Targeting apoptotic mechanisms in lung cancer can be a more effective therapy with fewer side effects since it will destruct cancer cells by itself without damaging other normal cells.

Berry volatiles are responsible for the berry aroma, but they have not received much attention compared to berry polyphenols [11,12]. Since berry volatiles were widely used in food, nutraceuticals, and cosmetic products, affecting fruit quality, fruit ripening, and fruit storage after harvest, perception, and acceptability of consumers, there have been many studies reporting the berry volatile compounds, but limited to identification, quantification, and sensory analyses [13,14,15]. However, recently there have been several studies that showed the health-beneficial effects of berry volatiles including anti-inflammatory activity [16]. Our recent study found that volatile extracts from six common berries (cranberry, black raspberry, red raspberry, strawberry, blueberry, and blackberry) showed an anti-inflammatory effect on lipopolysaccharide (LPS)-stimulated murine macrophage cells by suppressing pro-inflammatory cytokines such as interleukin-6 (IL-6) and tumor necrosis factor-α (TNF-α) via down-regulating nuclear factor-κB (NF-κB) signaling pathway [17]. Among six berries, blackberries and black raspberries showed the highest volatile content and blueberry volatile extract exhibited the greatest anti-inflammatory effect [17]. Blackberry volatile extracts with three different genotypes (A2528T, A2587T, and Natchez) also significantly attenuated pro-inflammatory cytokines [18]. Cranberry volatile extract and its major volatile compound α-terpineol significantly suppressed the level of nitric oxide (NO) in LPS-activated RAW264.7 macrophage cells [19]. Since berry volatiles can be absorbed into the lung through inhalation, and inflammation in the lung can damage the lung tissues and develop into lung cancer with high mortality, the objectives of this study were to evaluate the chemical compositions of volatile extracts from black raspberries, blueberries, and blackberries, and to investigate the antiproliferative effect of three berry volatile extracts on non-small-cell A549 lung cancer cells and their apoptotic mechanisms.

## 2. Materials and Methods

### 2.1. Materials

Frozen blueberries and blackberries were purchased from a local supermarket (Walmart, Fayetteville, AR, USA). Frozen black raspberries were obtained from Frank Farms (Eau Claire, MI, USA). Dulbecco’s modified Eagle’s medium (DMEM), fetal bovine serum (FBS), phosphate-buffered saline (PBS, pH 7.2), L-glutamine, penicillin and streptomycin, and other cell culture reagents were purchased from Thermo Fisher Scientific (Waltham, MA, USA). 3-(4,5-dimethylthiazol-2-yl)-5-(3 carboxymethoxyphenyl)-2-(4-sulfophenyl)2H-tetrazolium (MTS) was obtained from Promega Co. (Madison, WI, USA). Primary antibodies including rabbit anti-caspase-3 (#9662), rabbit anti-caspase-9 (#9502), rabbit anti-poly ADP-ribose polymerase (PARP, #9542), and rabbit anti-β-actin (#4970), and secondary antibody including goat anti-rabbit IgG, HRP-linked antibody (#7074) were purchased from Cell Signaling Technology, Inc. (Danvers, MA, USA).

### 2.2. Sample Preparation

Black raspberry, blueberry, and blackberry volatiles were extracted using hydrodistillation under low temperature and pressure as previously described [17]. Briefly, berries were blended with deionized water and sodium chloride (3:3:1 *w/w/w*) and vacuum distilled by a rotary evaporator (Buchi, Flawil, Switzerland) in a 50 °C water bath and 0 °C condenser for 30 min. Berry volatile extracts collected in an ice-bath-incubated flask were stored at −20 °C until use. An 85 μM, CAR/PDMS (carboxen^®^/polydimethylsiloxane), Stableflex, 24 Ga, Manual Supelco (Bellefonte, PA, USA) solid phase micro-extraction (SPME) fiber was used for the solid phase micro-extraction of volatiles from three berry volatile extracts, inserted into a vial containing berry volatile extract for 30 min at 65 °C by stirring.

### 2.3. GC/GC-MS Analysis of Berry Volatiles

Quantification and identification of berry volatiles using gas chromatography (GC)/GC-mass spectrometry (GC-MS) were conducted as previously described [19]. Briefly, volatiles absorbed to the SPME fibers were desorbed at 270 °C for 2 min in the injection port of a Varian 3800 GC (Agilent Technologies, Santa Clara, CA, USA) equipped with an HP-5 (5% phenyl-methylpolysiloxane) column (30 m × 0.25 mm, film thickness 1 μm) (Agilent Technologies, Santa Clara, CA, USA). The injection port was operated in splitless mode with a constant He flow of 25 psi. The initial oven temperature was 25 °C, held for 4 min, ramped at 12 °C/min to 289 °C, and held for 3 min. SPME-collected volatiles were analyzed by GC-MS using a Hewlett-Packard HP 5890 series gas chromatograph equipped with a mass selective detector (MSD) and an HP-5 capillary column (Agilent, 30 m × 0.25 mm, film thickness 1 μm). Volatiles were identified by comparing their mass spectra with the spectral library (Wiley7NIST0.5), literature data, and retention indices for standards.

### 2.4. Cell Culture and Berry Volatile Treatment

A human lung carcinoma cell line, A549 (ATCC^®^ CCL-185™), was purchased from American Type Culture Collection (ATCC, Manassas, VA, USA). Cells were cultured in DMEM supplemented with 10% FBS, 1% of 100 U/mL penicillin, and 100 μg/mL streptomycin. A549 cells were incubated at 37 °C in a humidified, 5% CO_2_ incubator (VWR^®^ Water Jacketed CO_2_ incubator, VWR International, PA, USA). For the assays, A549 cells were treated with 2, 4, and 8-fold diluted black raspberry volatile extract (BKV), blueberry volatile extract (BUV), or blackberry volatile extract (BKV) for 48 h. Tween80 (final concentration of 0.1%) was used to emulsify berry volatiles in the medium.

### 2.5. Cell Viability Assay

Cell viability of A549 lung cancer cells treated with BRV, BUV, and BKV was examined with the CellTiter 96^®^ AQueous One Solution Cell Proliferation Assay (MTS assay) (Promega Co., Madison, WI, USA) according to the manufacturer’s instruction. Briefly, cells were seeded onto a 96-well plate at a density of 1 × 10^4^ cells/well, and treated with 2, 4, and 8-fold diluted BRV, BUV, and BKV for 0, 12, 24, and 48 h. Cells were washed with PBS twice after treatment and 100 μL of culture medium was added to the cells. CellTiter 96^®^ AQueous One Solution Reagent (20 μL) containing the MTS tetrazolium compound was added to each well of the 96-well plate and incubated at 37 °C for 1 h in a humidified, 5% CO_2_ atmosphere. The MTS tetrazolium compound can be reduced into a colored formazan product by viable cells. Metabolically active cells use nicotinamide adenine dinucleotide (NADH)- or nicotinamide adenine dinucleotide phosphate (NADPH)-dependent dehydrogenase enzymes for the reduction. The absorbance at 490 nm was recorded by using a microplate reader (Synergy HT Multi-Mode Microplate Reader, BioTek Instruments, Inc., Winooski, VT, USA). The quantified amount of formazan product indicates the proportion of the number of viable cells with metabolic activity.

### 2.6. Apoptosis ELISA Assay

To evaluate the apoptotic effect of BRV, BUV, and BKV on A549 cells, cell death detection enzyme-linked immunosorbent assay (ELISA) kit (Roche, Indianapolis, IN, USA) was used. After 24 h incubation of A549 cells with 2-fold diluted three berry volatile extracts (BVEs), the enrichment of nucleosomes in the cytoplasm of cells was measured by using the cell death detection ELISA kit, according to the manufacturer’s instruction.

### 2.7. Flow Cytometry Analysis for Apoptosis

To investigate the rate of apoptotic lung cancer cells, fluorescein isothiocyanate (FITC) Annexin V Apoptosis Detection Kits (BD Biosciences, San Diego, CA, USA) were used. Cells were seeded onto 9 mm cell culture dishes and treated with 2-fold diluted three BVEs for 48 h. Cells were then collected, centrifuged at 280× *g* for 5 min, and pellets were washed with cold PBS twice. The pellets were resuspended in a binding buffer. In 100 μL of the solution, 5 μL of FITC Annexin V and 5 μL of propidium iodide (PI) were added and incubated for 15 min in the dark. After adding 400 μL of binding buffer, samples were analyzed by using a flow cytometer (BD FACS Aria Fusion, BD FACS Aria III, BD Biosciences, San Diego, CA, USA) to measure the rate of cell apoptosis (FITC+/PI- and FITC+/PI+).

### 2.8. Cell Cycle Analysis

To assess the effect of three BVEs on the cell cycle progression, A549 cells were treated with 2-fold diluted three BVEs and fixed with 70% ethanol for 2 h. After washing with cold PBS twice, 500 μL of PI/RNase staining buffer (BD Biosciences, San Diego, CA, USA) was added and incubated for 15 min. Fluorescence-activated cell sorting cytometer (BD FACS Aria Fusion, BD Biosciences, San Diego, CA, USA) was used to analyze cell cycle progression.

### 2.9. Western Blot Analysis

Cells were seeded onto a 9 mm cell culture dish at a density of 10 × 10^4^ cells/mL, containing 10 mL of medium, and incubated at 37 °C in a 5% CO_2_ incubator. After overnight, cells were treated with 2-fold diluted three BVEs for 48 h, and then cells were collected. The collected cell suspension was centrifuged at 280× *g* for 5 min, and pellets were washed twice with cold PBS, repeating centrifugation. The cells were lysed by using a lysis buffer (radioimmunoprecipitation assay (RIPA) buffer, MilliporeSigma Corporate, St. Louis, MO, USA) for 1 h on ice. After centrifugation at 9240× *g* for 15 min at 4 °C, supernatants were used to measure the concentration of protein in samples by using bicinchoninic acid (BCA) protein assay kit (Thermo Fisher Scientific, Waltham, MA, USA). Samples with 40 μg of protein were separated on 4–20% sodium dodecyl-sulfate (SDS)-polyacrylamide gel and transferred onto a polyvinylidene fluoride (PVDF) membrane (Bio-Rad Laboratories, Inc., Hercules, CA, USA) with a glycine transfer buffer (25 mM Tris, 192 mM glycine, pH 8.3). The non-specific sites on the membrane were blocked by using 3% bovine serum albumin (BSA) and the membrane was incubated overnight with primary antibodies (1:5000 for β-actin and 1:1000 for the others) at 4 °C. After overnight incubation, the membrane was washed with tris-buffered saline containing 0.1% Tween20 (TBST) three times for 30 min and incubated with horseradish peroxidase (HRP)-linked antibody (Cell Signaling Technology, Inc., Danvers, MA, USA) for 1 h. After washing with TBST three times, the protein bands were developed using an enhanced chemiluminescent (ECL) Western blotting detection kit (Bio-Rad Laboratories, Inc., Hercules, CA, USA) and captured and developed on x-ray film (CL-XPosure™ Film, Thermo Fisher Scientific, Waltham, MA, USA) in a dark room. The density of the bands was analyzed by using ImageJ software (Version: 1.52a, U.S. National Institutes of Health, Bethesda, MD, USA).

### 2.10. Statistical Analysis

SAS 9.4 (SAS Institute Inc., Cary, NC, USA) was used to perform all statistical analyses. Data were presented as the mean ± standard error of the mean (SEM). All experiments were conducted in at least triplicate. Significant differences among the treatments were determined using a one-way analysis of variance (ANOVA) and Tukey’s test at 5% level of significance.

## 3. Results

### 3.1. Chemical Composition of Berry Volatile Extracts

**Figure 1:**
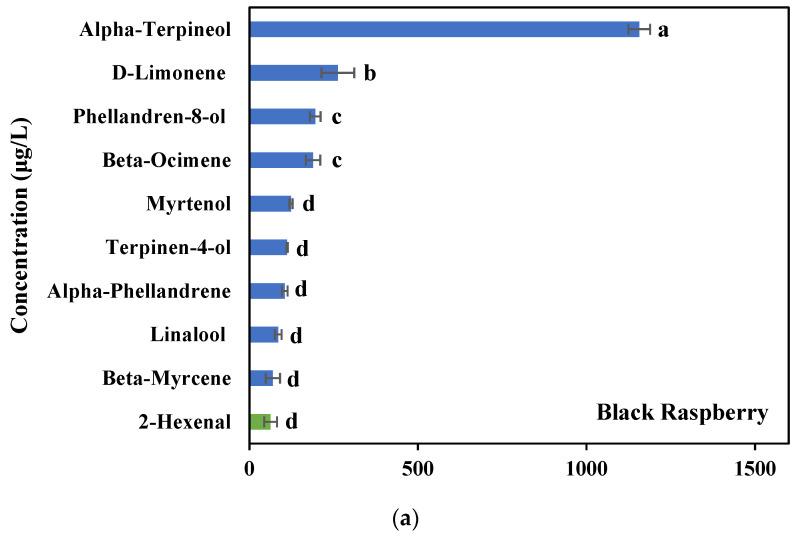

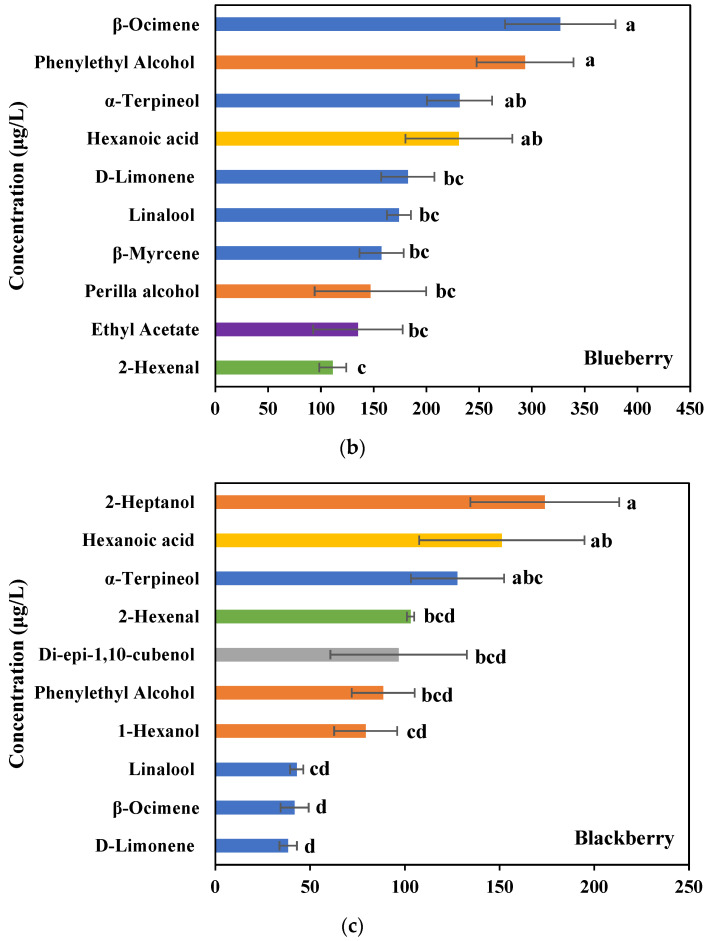
Ten major volatile compounds identified from (**a**) black raspberry, (**b**) blueberry, and (**c**) blackberry volatile extracts. Data are expressed as the mean ± SD (*n* = 3). Means followed by a common letter are not significantly different at the 5% level. Bar color yellow = acid; orange = alcohol; green = aldehyde; purple = ester; blue = monoterpene; gray = sesquiterpene.

Total volatiles in each berry extract were presented in Table 1, with the content of volatile classes in each berry extract. The volatile composition of three berry volatile extracts was determined by using GC/GC-MS analysis and ten major individual volatile compounds identified from three BVEs were presented in Figure 1.

The total volatile content of black raspberry, blueberry, and blackberry volatile extracts were 3.2 ± 0.1 mg/L, 3.1 ± 0.3 mg/L, and 1.6 ± 0.2 mg/L, respectively (Table 1). Black raspberry showed the highest volatile content, followed by blueberry and blackberry. Among the volatile classes, monoterpene was one of the most abundant volatiles identified in all three berry volatile extracts, as 2.7 ± 0.1 mg/L in BRV, 1.5 ± 0.2 mg/L in BUV, and 0.5 ± 0.1 mg/L in BKV. Alcohol showed the second most abundant volatiles in both BUV (0.7 ± 0.1 mg/L) and BKV (0.5 ± 0.0 mg/L), while aldehyde was the second most abundant in BRV (0.2 ± 0.0 mg/L). At the individual level of volatiles, the highest individual volatile compound in each berry volatile extract was α-terpineol in black raspberries, β-ocimene in blueberries, and 2-heptanol in blackberries (Figure 1). Major volatile compounds identified from all three berry volatile extracts were α-terpineol, β-ocimene, d-limonene, linalool, and 2-hexenal.

### 3.2. Effect of Berry Volatile Extracts on A549 Cell Viability

**Figure 2:**
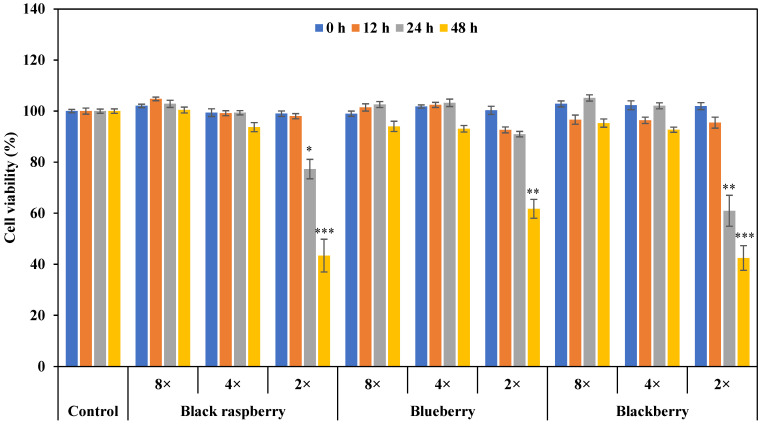
The effect of berry volatiles (2, 4, 8-fold dilution) on the proliferation of A549 cells. Data are expressed as the mean ± SEM (*n* = 15). Significant differences are compared with control at * *p* < 0.05, ** *p* < 0.01, and *** *p* < 0.001.

To examine the effect of three berry volatile extracts on lung cancer cells, A549 cells were treated with three different dilutions (2, 4, and 8-fold) of three berry volatile extracts (black raspberry, blueberry, and blackberry) for 0, 12, 24, and 48 h. Tween 80 (0.1% final concentration) was used to emulsify berry volatile extracts in a culture medium. The cytotoxicity of Tween 80 itself on A549 cells was preliminarily tested, and Tween 80 at the concentration of 0.1% or below showed no cytotoxic effect (data not shown). The viability of A549 decreased gradually in a time-dependent manner with the 2-fold diluted berry volatile extracts treatment (Figure 2). Black raspberry and blackberry volatile extracts with 2-fold dilution significantly inhibited the A549 cell proliferation after 24 h of treatment (*p* < 0.05). All groups with 2-fold diluted berry volatile extract treatments showed a significant decrease in lung cancer cell viability compared to the control after 48 h of treatment (*p* < 0.01). The IC50 (the half maximum inhibitory concentration) of black raspberry, blueberry, and blackberry volatile extracts at 48 h were 1.5 mg/L, 2.0 mg/L, and 0.7 mg/L for A549 cells, respectively. Among three berries, blackberry and black raspberry volatile extracts showed stronger suppression in A549 viability compared to blueberry when treated with 2-fold dilution for 48 h (*p* < 0.05). It showed that 2-fold diluted berry volatile extracts significantly suppress the proliferation of lung cancer cells. Therefore, a 2-fold dilution of berry volatile extracts was used for further assays.

### 3.3. Apoptosis ELISA Assay

**Figure 3:**
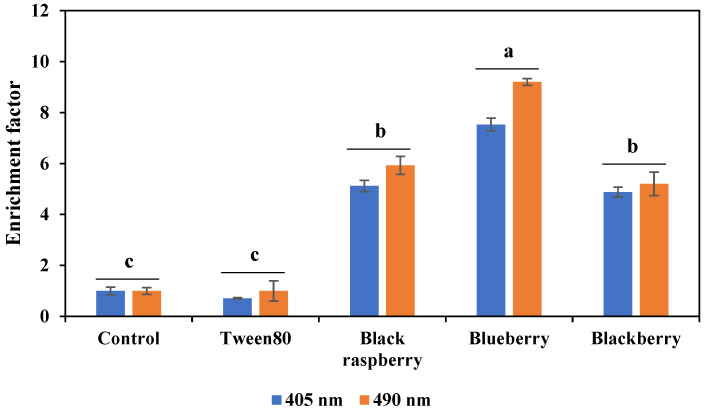
Berry volatiles induced apoptosis in human non-small-cell lung cancer A549 cells as measured by ELISA assay. A549 cells were treated with black raspberry, blueberry, and blackberry volatile extracts for 24 h at 2-fold dilution. Data are expressed as the mean ± SEM (*n* = 3). Means followed by a common letter are not significantly different at the 5% level.

To investigate whether the antiproliferative effect of berry volatile extracts on A549 cells was related to the apoptosis in the cells, the effect of berry volatile extracts on apoptosis in A549 cells was examined by using a cell death detection ELISA kit (Roche, Indianapolis, IN, USA). In Figure 3, cells treated with only 0.1% Tween80 did not show much difference compared to the control. However, cells treated with 2-fold diluted three berry volatile extracts (BRV, BUV, and BKV) for 24 h showed significantly increased nucleosomes in the cytoplasm of cells compared to the control (*p* < 0.05), indicating one of the characteristics of apoptotic cells, DNA fragmentation. Among three berries, blueberry volatile extracts showed the strongest apoptotic effect in A549 cells compared to the other two berries (*p* < 0.05). These results indicate that three berry volatile extracts have an antiproliferative effect on A549 lung cancer cells partially by inducing apoptosis.

### 3.4. Flow Cytometric Analysis for Apoptosis

**Figure 4:**
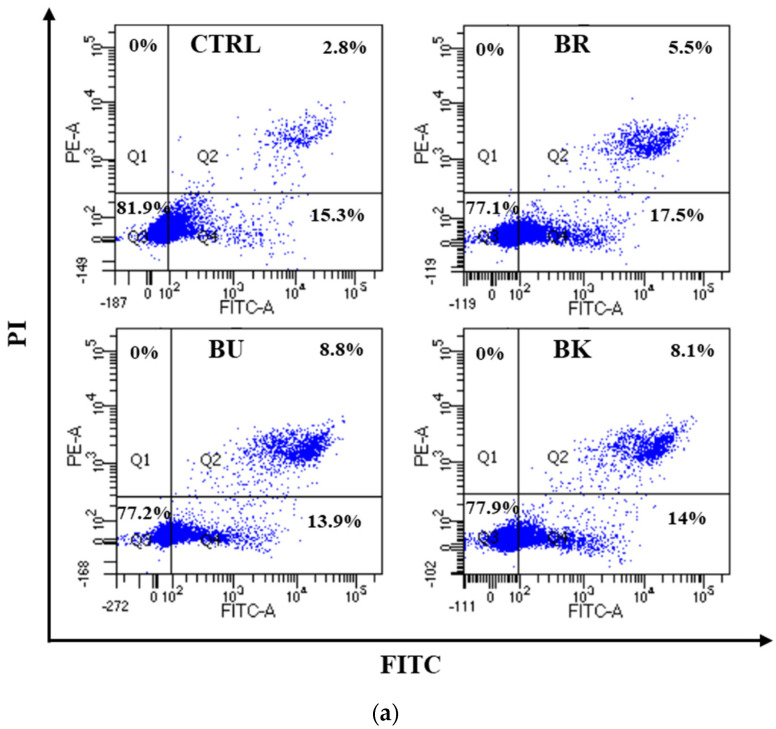

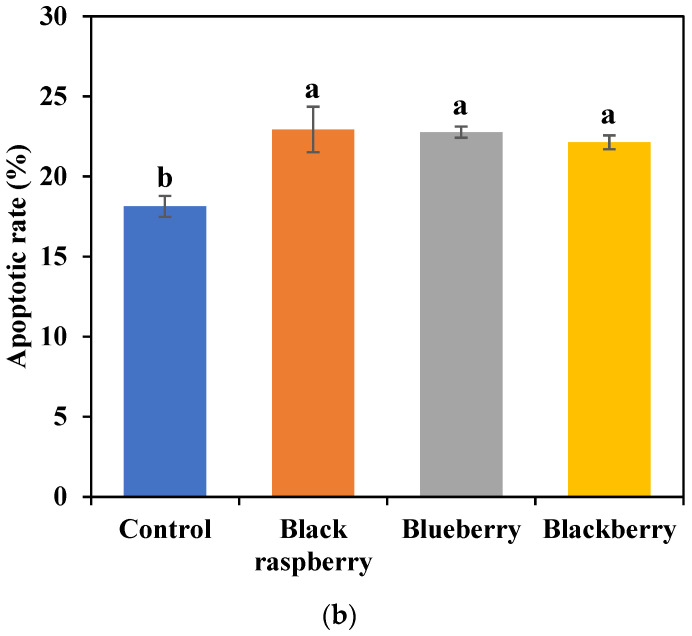
(**a**) Flow cytometric analysis of Annexin V-FITC/PI stained A549 cells and (**b**) the apoptotic rates (%) of three BVE-treated A549 cells. A549 cells were treated with BR, BU, and BK volatile extracts for 48 h at 2-fold dilution. Data are expressed as the mean ± SEM (*n* = 3). Q1 = necrosis; Q2 = late apoptosis; Q3 = viable cells; Q4 = early apoptosis; FITC = fluorescein isothiocyanate; PI = propidium iodide; CTRL = control; BR = black raspberry; BU = blueberry; BK = blackberry; BVE = berry volatile extract. Means followed by a common letter are not significantly different at the 5% level.

To examine the apoptotic rate of cells when treated with berry volatile extracts, flow cytometric analysis for apoptosis was performed. As shown in Figure 4, the apoptotic rates of cells in groups (early and late apoptosis) were 18.1 ± 0.7% (CTRL), 22.9 ± 1.4% (BR), 22.8 ± 0.3% (BU), and 22.1 ± 0.04% (BK). A549 cells treated with 2-fold diluted all three berry volatile extracts (BRV, BUV, and BKV) showed significantly increased apoptotic rates compared to the control (*p* < 0.05).

### 3.5. Cell Cycle Analysis

**Figure 5:**
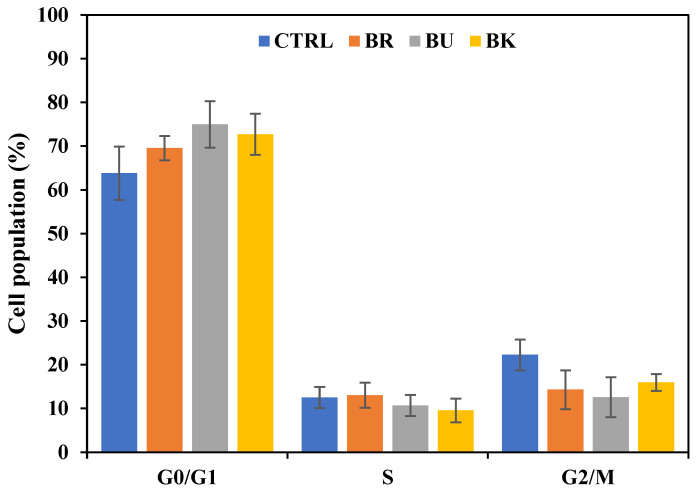
The effect of berry volatile extracts on cell cycle progression measured by flow cytometric analysis of PI-stained A549 cells. A549 cells were treated with BRV, BUV, and BKV for 48 h at 2-fold dilution. Data are expressed as the mean ± SEM (*n* = 3).

Since the cell cycle is related to apoptosis, the effect of three berry volatile extracts on A549 cell cycle progression was investigated by using a flow cytometer. As a result, the cell population in G0/G1 phase slightly increased in BRV (69.6 ± 2.8%), BUV (75.0 ± 5.3%), and BKV (72.7 ± 4.7%) compared to the control (63.8 ± 6.1%) (Figure 5). While the cell population in G0/G1 phase slightly increased, the ones in G2/M phase slightly decreased in BRV (14.3 ± 4.4%), BUV (12.6 ± 4.6%), and BKV (16.0 ± 1.9%) compared to the control (22.3 ± 3.5%). These results indicate that berry volatile extracts may suppress the cell cycle progression at G0/G1 phase and increase apoptosis.

### 3.6. Western Blot Analysis

**Figure 6:**
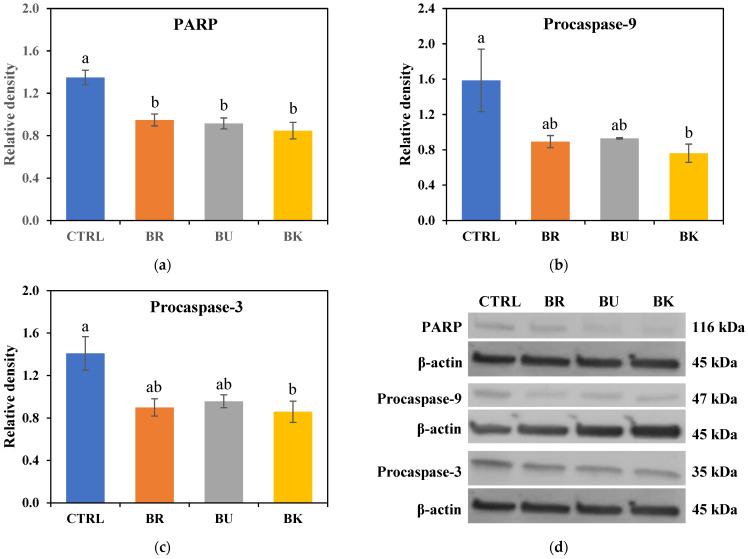
The effect of berry volatile extracts on protein expression levels of (**a**) PARP, (**b**) procaspase-9, and (**c**) procaspase-3 by Western blot analysis of A549 cells and (**d**) representative Western blots of PARP, procaspase-9, procaspase-3, and β-actin. A549 cells were treated with BR, BU, and BK volatile extracts for 48 h at 2-fold dilution. Data are expressed as the mean ± SEM (*n* = 3). PARP = poly adenosine diphosphate-ribose polymerase; CTRL = control; BR = black raspberry; BU = blueberry; BK = blackberry. Means followed by a common letter are not significantly different at the 5% level.

To further investigate the effect of berry volatile extracts on apoptosis-related protein expression in A549 cells, the levels of apoptosis-regulating proteins were measured by using Western blot analysis. In Figure 6, the level of PARP was significantly decreased by all three berry volatile extracts with 2-fold dilution (*p* < 0.05). Two-fold diluted BKV significantly reduced the level of procaspase-9 and procaspase-3 (*p* < 0.05). Two-fold diluted BRV and BUV did not show a significant difference but slightly decreased procaspase-9 and procaspase-3.

## 4. Discussion

Essential oils from plants containing various volatiles have been reported to have many health effects including anti-cancer, anti-inflammatory, antioxidant, and antimicrobial activities [20,21,22]. Especially, monoterpenes are one of the most predominant compounds in essential oils [23]. Monoterpenes are aromatic 10C isoprenoids and are widely used in the food and cosmetic industry due to their aromatic characteristics [23]. There have been many studies on the antitumor activities of monoterpenes [16,24,25,26]. Berries also contain lots of volatile compounds that are found in other plant essential oils, but the health effects of berry volatiles have not been extensively studied. Thus, in this study, the chemical composition of black raspberry, blueberry, and blackberry volatile extracts and their antiproliferative effects on lung cancer cells through apoptotic mechanisms were investigated. In the current study of the chemical composition of berry volatiles from black raspberries, blueberries, and blackberries, monoterpenes were determined as the major compound. Major monoterpenes found in all three berry volatile extracts were α-terpineol, β-ocimene, d-limonene, and linalool (Figure 1). α-terpineol can be found in many natural sources such as essential oils of fruits, flowers, herbs, and others [27]. Linalool is also one of the major compounds in plant oils, belonging to more than 50% of plant families [28]. These major compounds have been suggested as natural alternative therapeutic agents with the potential for antioxidant, anti-inflammatory, antiproliferative, antimicrobial, and anti-cancer effects [29,30,31].

The current study demonstrated that three berry volatile extracts significantly suppressed the proliferation of A549 lung cancer cells with an increase in cell apoptosis. Apoptosis causes cell shrinkage, nuclear fragmentation, chromatin condensation, chromosomal DNA fragmentation, and eventually death [32]. The level of DNA fragmentation of berry volatile extract-treated lung cancer cells was examined using cell death detection ELISA assay. The results showed that black raspberry, blueberry, and blackberry volatile extracts showed a significant increase in apoptosis with increased nucleosomes in the cytoplasm of the cells after 24 h treatment. Apoptosis in A549 cells was also assessed using flow cytometry. Black raspberry, blueberry, and blackberry volatile extracts with 2-fold dilution significantly increased the apoptotic rate of A549 cells (*p* < 0.05). Linalool (1 and 2 mM) and 1,8-cineole (4 and 8 mM) showed a significant decrease in A549 lung cancer cell proliferation [33]. Linalool (2 mM) slightly increased the apoptotic cell death, but 1,8-cineole did not show an apoptotic effect. In Bai and Tang [34], myrcene (0.25–1 μg/mL) showed a significant cytotoxic effect in A549 lung cancer cells after 48 h. Essential oils from *Pittosporum tobira* flowers showed a significant antiproliferative effect on A549 cells [35]. In essential oils from *Pittosporum tobira* flowers, the main compounds were α-pinene, β-myrcene, γ-elemene, β-ocimene, d-limonene, and nerolidol.

The cell cycle is closely related to apoptosis [36]. Each cell has its cell cycle, and during the cycle, if the damage cannot be repaired in cells, then it goes to G0/G1 phase where apoptosis occurs. Apoptosis is mediated by enzymes, called caspases [7]. Caspases trigger cell death by cleaving apoptotic proteins. Caspases exist in all cells as inactive forms, procaspases. They are activated by the cleavage of other caspases, producing a cascade of activation. Poly adenosine diphosphate (ADP)-ribose polymerase (PARP) is another critical enzyme related to DNA repair [37]. The current study showed a slight increase in the G0/G1 phase of lung cancer cells with the treatment of three berry volatile extracts for 48 h. In this study, three berry volatile extracts decreased the level of inactive form of caspase-3 and 9, and PARP, especially with blackberry volatile extracts significantly down-regulating procaspase-3 and 9, and PARP (*p* < 0.05). Black raspberry and blueberry volatile extracts significantly suppressed the level of PARP (*p* < 0.05). Rodenak-Kladniew et al. [33] found that both linalool and 1,8-cineole increased G0/G1 phase arrest. However, there were no changes in the level of cleaved caspase-3, procaspase-9, and PARP levels with linalool or 1,8-cineole treatment. Myrcene with a concentration of 1 μg/mL showed strong cell cycle arrest at G0/G1 phase and significantly increased the expression level of caspase-3, caspase-9, and cytochrome C in A549 lung cancer cells [34]. In Sun et al. [35], essential oils from *Pittosporum tobira* flowers decreased cell population in G0/G1 phase and increased in G2/M phase in A549 cells.

From these results, the current study demonstrated that volatile extracts from blackberry, black raspberry, and blueberry have an antiproliferative effect on lung cancer cells in vitro and suggested that it is partially due to cell apoptosis. However, since this study was conducted with the whole berry volatile extracts, more extensive research with individual volatile compounds in berries and in vivo studies will be needed.

## 5. Conclusions

In this study, it was demonstrated that blackberry, black raspberry, and blueberry volatile extracts have an antiproliferative effect against non-small cell lung carcinoma cells. Furthermore, we found that the antiproliferative effect of berry volatile extracts may be due to the increase in cell apoptosis by down-regulating the expression level of procaspase-3 and 9, and PARP. This suggests that berry volatiles from blackberry, black raspberry, and blueberry may have potential anti-cancer activity through apoptosis in lung cancer.

## Figures and Tables

**Table 1:** Chemical Composition of Volatile Extracts from Black Raspberry, Blueberry, and Blackberry.

Class	Black Raspberry	Blueberry	Blackberry
	(μg/L)		
**Acid**	81.3 ± 22.63 ^Cc^ *	303.1 ± 40.8 ^Ac^	182.8 ± 45.8 ^Bb^
**Alcohol**	190.9 ± 5.5 ^Cb^	733.0 ± 97.5 ^Ab^	463.1 ± 43.5 ^Ba^
**Aldehyde**	203.0 ± 31.9 ^b^	203.1 ± 5.1 ^cd^	173.0 ± 6.5 ^b^
**Ester**	13.8 ± 0.5 ^Bd^	188.8 ± 41.5 ^Acde^	43.0 ± 14.8 ^Bc^
**Hydrocarbon**	8.1 ± 2.3 ^Ad^	0.4 ± 0.0 ^Be^	2.8 ± 1.0 ^Bc^
**Ketone**	10.9 ± 4.4 ^Ad^	4.4 ± 0.1 ^Be^	3.2 ± 0.5 ^Bc^
**Monoterpene**	2659.5 ± 52.5 ^Aa^	1515.5 ± 177.4 ^Ba^	505.3 ± 67.8 ^Ca^
**Norisoprenoid**	0.1 ± 0.0 ^Cd^	0.7 ± 0.1 ^Be^	1.1 ± 0.1 ^Ac^
**Sesquiterpene**	1.7 ± 0.5 ^Bd^	157.1 ± 43.6 ^Acde^	175.1 ± 73.1 ^Ab^
**Others**	1.5 ± 0.4 ^Cd^	15.56 ± 2.6 ^Bde^	39.1 ± 7.1 ^Ac^
**Total**	3184.9 ± 75.3 ^A^	3107.5 ± 256.4 ^A^	1588.5 ± 170.9 ^B^

* Values within rows with different capital letters, and values within columns with different lowercase letters are significantly different (*p* < 0.05). Data are expressed as the mean ± standard deviation (SD) (*n* = 3).

## Data Availability

Not applicable.
